# Extracorporeal membrane oxygenation support and total thyroidectomy in patients with refractory thyroid storm: case series and literature review

**DOI:** 10.1093/jscr/rjac131

**Published:** 2022-05-17

**Authors:** Murad Alahmad, Maryam Al-Sulaiti, Husham Abdelrahman, Ayman El-Menyar, Inaita Singh, Abdelhakeem Tabeb, Hassan Al-Thani

**Affiliations:** Department of Surgery, General Surgery, Hamad Medical Corporation, Qatar; Department of Surgery, General Surgery, Hamad Medical Corporation, Qatar; Department of Surgery, Trauma Surgery, Hamad Medical Corporation, Qatar; Department of Surgery, Trauma Surgery, Clinical Research, Hamad Medical Corporation, Qatar; Clinical Medicine, Weill Cornell Medical College, Doha, Qatar; Medical Education, Internship, Hamad Medical Corporation, Qatar; Department of Surgery, General Surgery, Hamad Medical Corporation, Qatar; Department of Surgery, Trauma and Vascular Surgery, Hamad Medical Corporation, Qatar

## Abstract

Thyroid storm (TS) is a rare but life-threatening complication of hyperthyroidism in which multiorgan failure (MOF) is the most common cause of death. Early diagnosis and treatment of TS are challenging. We presented two cases with refractory TS complicated with arrhythmia, cardiac arrest, cardiogenic shock and MOF and were not responding to medical treatment, therapeutic plasma exchange or continuous renal replacement therapy. The combination of extracorporeal membrane oxygenation (ECMO) and early thyroidectomy was the mainstay treatment that was performed with no complications. MOF was resolved and patients were doing well in the outpatient clinic follow-up. Precautions concerning the beta blockers and anti-thyroid medications use in TS, especially in the acute setting, should be considered. Upon its availability, the use of ECMO and early thyroidectomy is efficient. This is most applicable in patients not responding to medical treatment or patients who develop complications related to the TS and its medical treatment.

## INTRODUCTION

Thyroid storm (TS) is a real endocrine emergency and a life-threatening complication that represents thyrotoxicosis extreme manifestations [1]. The first description was by Lahey in 1926 and until now, it represents a diagnostic and therapeutic challenge [2]. The clinical presentation is extremely variable. It includes fever, tachycardia, abdominal pain, tachyarrhythmia, multiorgan failure (MOF) and circulatory collapse [3].

The TS incidence ranges from 0.57 to 0.76 per 100,000 per year in the general population and 4.8 to 5.6 cases/100,000 per year in the hospitalized patients [4]. The mainstay treatment is medical including a combination of beta-blockers and antithyroid medications [5]. However, failure of medical treatment calls for other interventions like therapeutic plasma exchange (TPE, plasmapheresis) and surgical ablation. Generally, TS contraindicates surgery [6]. Herein, we present two refractory TS cases complicated with MOF, where extracorporeal membrane oxygenation (ECMO) support and total thyroidectomy were performed successfully. In this case series, ECMO acted as a safety bridge for surgery. This report was given according to the care report guidelines (CARE checklist) (Supplementary Table).

## CASE PRESENTATION

**Figure 1:**
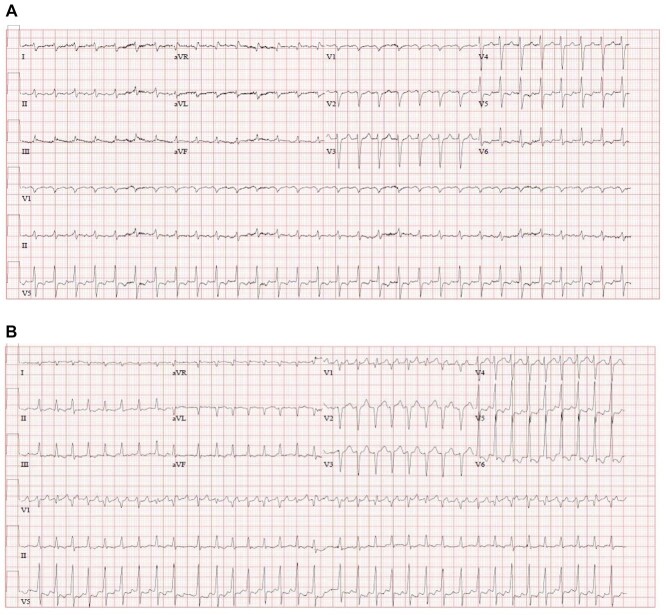
(**A**) ECG on admission (case 1). (**B**) ECG on admission (case 2).

Case 1: a 46-year-old female patient, known to have primary thyrotoxicosis, but she was not compliant with her medications. She presented to the emergency department (ED) with epigastric pain and vomiting for 2 hours. Grave’s disease signs (proptosis, lid lag and retraction, and tremors) were apparent. While in the ED, she started to have palpations and shortness of breath. Her electrocardiogram (ECG) showed atrial fibrillation with a heart rate of 200 beats per minute (Fig. 1A).

**Figure 2:**
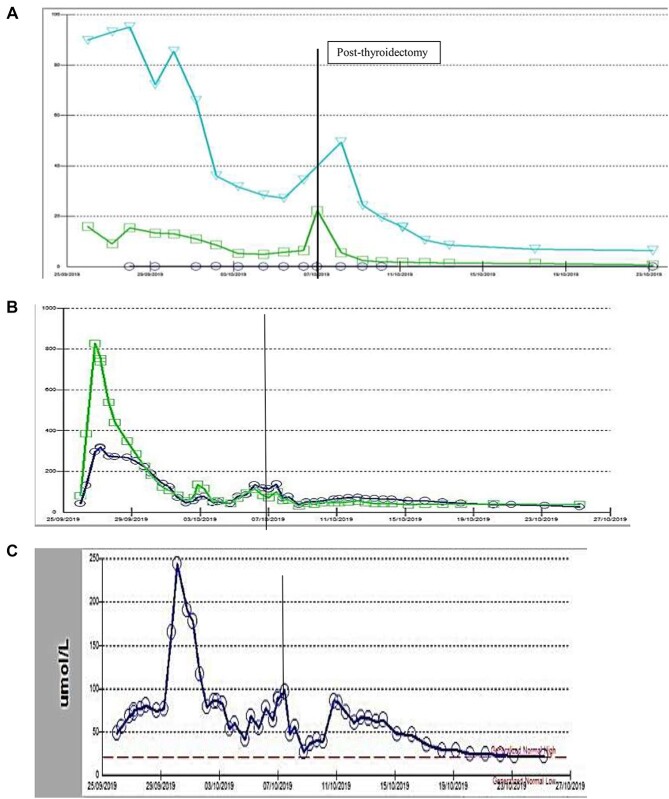
(**A**) Thyroid function test (case-1): circle: TSH, square: FT3, triangle: FT4, (**B**) Liver function tests (case-1): circle: ALT, square: AST, (**C**) Liver function tests (case-1): bilirubin level, vertical line: date of surgery.

Laboratory results were reflecting a picture of MOF including acute kidney injury (AKI), liver failure with elevated aminotransferase/alanine aminotransferase (AST/ALT), INR and Bilirubin as shown in Table 1. Thyroid function test parameters showed extremely low thyroid-stimulating hormone <0.01 MIU/L, high T3/T4 (16 and 19 pmol/L, respectively) and increased lactic acid 10 mmol/L (Fig. 2). Thyroid storm was the provisional diagnosis. The team started intravenous beta blocker (propranolol), hydrocortisone and oral propyl thiouracil. However, she became progressively tachypnic, and restless with severe desaturation and respiratory failure. This clinical deterioration demanded intubation and transfer to the Medical Intensive Care Unit (MICU) due to TS with progressively failing organs.

**Table 1:** The laboratory results in the two cases before and after surgery

Blood test	Normal value/unit	First reading		Before surgery		After surgery	
		Case1	Case2	Case1	Case2	Case1	Case2
Creatinine	62–106 μmol/L	76	135	42	98	30	49
AST	0–39 U/L	81	195	114	477	51	25
ALT	5–30 U/L	42	498	134	393	31	14
Bilirubin	0–21 μmol/L	70	140	80	345	17	25
Serum lactate	0.5–2.2 mmol/L	10	11.6	13	n/a	n/a	n/a
T3	3.7–6.4 pmol/L	15.9	4.9	6.4	3.2	1.6	2.2
T4	11.6–21.9 pmol/L	89.9	40	34	30.6	9.8	15.2
TSH	0.3–4.2 mIU/L	<0.01	<0.01	0.02	0.03	0.36	6.4
Hs–Troponin	1–14 ng/L	33	350	45.8	74	83.4	32
INR	1	1.9	3.4	1.4	1.8	1.4	1.4

On arrival to the MICU, she was hypotensive and in shock with a blood pressure of 60/34, and arterial blood gas showed high lactate 13 mmol/L. She was in pulmonary edema as well. Bedside echocardiography (ECHO) showed severe left and right ventricular dysfunction with an ejection fraction of 24%. Despite the inotropes support (Dobutamine, Adrenalin and Noradrenalin), the blood pressure was not responding. Her progressively worsening condition necessitated a venous–arterial extracorporeal membrane oxygenation (ECMO) support. The patient cardiac function and vitals improved on ECMO with a left ventricular ejection fraction of 35% and was weaned off after five days.

During that time, her liver enzymes remained high due to liver shock and antithyroid medications. The team had to stop antithyroid medications and initiated therapeutic plasma exchange (TPE, plasmapheresis). One day after weaning off the ECMO, a follow-up ECHO showed left atrium and inferior vena cava thrombosis that could be related to ECMO catheter and her liver enzymes were still high despite holding antithyroid medications and heparin infusion. The patient course got more complicated, and the multi-disciplinary team discussion was raised and deemed the case as refractory TS. Surgery was considered as the last resort to control the medical condition. The surgical team planned for total thyroidectomy understanding the critical and complex situation. The patient was under sedation, on heparin infusion, with low cardiac ejection fraction, high cardiac marker (Hs-Troponin) and acute liver injury. Nevertheless, the patient underwent an uneventful total thyroidectomy, tolerated the surgery well, and shifted back to the Intensive Care Unit. Interestingly, after surgery, the patient’s laboratory results started to improve and gradually all her organ functions recovered (Table 1). The patient went home after one month. On outpatient clinic follow-up, the patient was doing fine with no active complaints.

Case 2: a 42-year-old female patient, with an unremarkable past medical history, was brought to the ED with palpitations, shortness of breath and abdominal pain. In the ED, initial resuscitation was initiated. ECG showed atrial flutter with a heart rate of 170 beats per minute and a blood pressure of 75/50 mmHg (Fig. 1B). Immediate synchronized cardioversion was performed. She went into ventricular tachycardia and was shocked again. While in the ED, the patient was given 2 mg of propranolol intravenously. Suddenly, the patient developed a seizure and cardiac arrest with pulseless electrical activity. Cardiopulmonary Resuscitation was performed for ten minutes including intubation as per Advanced Cardiovascular life support protocol and return of Spontaneous Circulation was achieved. The patient arrived the MICU with a blood pressure of 80/60 mmHg. Bedside ECHO demonstrated poor ejection fraction of 14%. Laboratory investigations revealed a markedly low thyroid stimulating hormone level of <0.01 pmol/L and elevated Free T4 levels of 38.9 pmol/L (Table 1). The patient’s skin was cold and clammy; phenylephrine and noradrenaline were given at 9 mcg/kg/min and 0.5 mcg/kg/min, respectively. Exophthalmos and jaundice were observed. TS complicated by cardiogenic shock and MOF was the provisional diagnosis. The patient was started on cholestyramine and 100 mg intravenous hydrocortisone. However, she remained in a state of shock.

**Figure 3:**
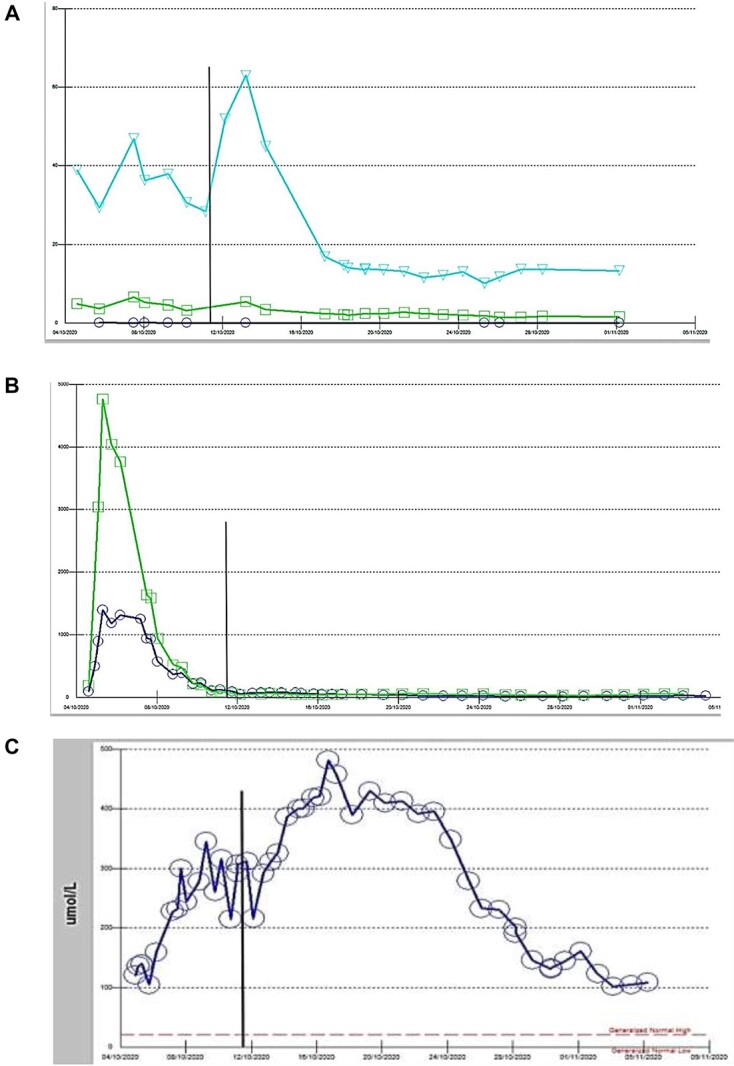
(**A**) Thyroid function test case 2: circle: TSH, square: FT3, triangle: FT4, vertical line: date of thyroidectomy, (**B**): Liver function test case 2: square: AST, circle: ALT, (**C**) Liver function test case 2: bilirubin.

Laboratory parameters were consistent with MOF, including liver failure and acute kidney injury with metabolic acidosis (Fig. 3). Rising high-sensitive Troponin T was also noted.

Due to liver failure, carbimazole and propylthiouracil were not given and the team initiated TPE. The patient’s clinical course was deteriorating with persistent hypotension despite of two inotropic agents. ECMO support was initiated for the patient’s refractory cardiogenic shock. The patient tolerated ECMO well with a significant improvement in her Ejection Fraction from 14% to 35% and she was weaned from ECMO within six days. Heparin was started after echocardiography that revealed intracardiac thrombi. Inotropes were tapered down, and her atrial fibrillation was medically controlled; however, her Free T4 levels remained high. The patient’s condition necessitated continuous renal replacement therapy.

Despite the medical treatment, the patient’s clinical status was extremely poor, and the decision to proceed with surgical intervention as the ultimate treatment was made. A total thyroidectomy was planned. Nevertheless, total thyroidectomy followed a smooth and uncomplicated course. Following surgery, the patient’s overall condition improved dramatically. Her ejection fraction rose to 40%, and vasopressors were discontinued. AST and ALT normalized and TSH improved. Hyperbilirubinemia improved gradually after surgery and reached the normal values after one month (Fig. 3). The patient had a steady recovery and was discharged home in the following month. Upon post-operative outpatient clinic follow up, the patient was doing well.

## DISCUSSION

The diagnosis of TS in this series was based on the Burch-Wartofsky Point Scale (>45) in two female patients. TS is a rare, life-threatening disorder characterized by multiorgan involvement and high mortality rate. It is three to five times more common in women, and the overall mortality can reach up to 10–30% [2, 7].

In the outpatient settings, noncompliance with antithyroid medications was the responsible insult and accounted for about 35% of reported TS cases; on the other hand, infection was the leading insulting event in the inpatient group [2, 6]. However, the precipitating factor was not clear in the second case.

The diagnosis of TS is challenging. It depends mainly on the clinical presentation and physician suspicion. There is no specific lab test to detect ongoing TS and no thyroid hormone level cutoff that differentiates patients with ‘uncomplicated’ thyrotoxicosis and TS. However, with high clinical suspicion of TS, treatment should be initiated immediately. The delay in the treatment can raise the mortality rate to 75% [2]. The most common causes of death in TS were multiple organ failure (liver, heart and lung) and arrhythmia [8]. Given its unpredictable course, treatment must be timely initiated [9]. The ultimate goals in treating TS are to decrease thyroid hormone synthesis, release, and the peripheral adrenergic effects of thyroid hormones. The medical treatment combining antithyroid medications and beta-blockers (BB) usually achieves these goals [10]. Nevertheless, BB must be given cautiously and selectively case by case as they can precipitate acute deterioration. In this series, after giving BB, the patients hemodynamics had deteriorated. Therefore, short-acting BB as esmolol may act better. In addition to one case report, Abubaker and colleagues summarized additional nine cases that developed propranolol-induced cardiovascular collapse in patients with thyroid crisis [11]. That is why it is recommended to use short-acting BB along with close hemodynamic monitoring and to be administered selectively case by case. The deterioration after BB could be explained by the exaggerated response to BB due to possible subclinical thyrocardiac disease, inhibition of the compensatory capacity of coronary vasodilatation and inhibition of the compensatory role (thyroid-induced hyperadrenergic status) in maintaining the cardiac output [11]. The TS-induced hyperadrenergic status could explain the arrhythmia, troponin release and low ejection fraction in our cases.

Surgical ablation, although it is the ultimate source control but, the frailty of patients with refractory thyroid storm makes surgery formidable. However, with the development of new advances in life support, the equation may change.

The current cases represent refractory TS with circulatory collapse, where ECMO allowed favorable surgical intervention. ECMO is an advanced life support machine. It can replace the heart and lungs’ function. It is used as a salvage therapy in cardiogenic shock and is becoming integrated with critical care support. Nevertheless, to date, only a few cases of its use in endocrine emergencies have been reported in the literature. We believe that the use of ECMO in those two cases of refractory TS significantly impacted the disease’s progress and allowed safe surgery. After initiation of ECMO support in both cases, the EF improved and allowed weaning off the inotropes by day five. It also optimized the patients’ condition before surgery and prevented the expected mortality. A case series by Chao A. et al. showed that ECMO use in 12 cases with refractory shock in endocrine emergencies, including five cases with TS, had even better outcome than its use in acute myocardial infarction patients. However, in that study, thyroid surgery was not performed for TS. [12]. Moreover, TS patients were initially misdiagnosed to have myocardial infarction and CHF and two of them died with MOF even after ECMO support [12].

Thyroidectomy in TS patient is a risky option with an overall mortality rate of 10%; that is why surgery is not the first choice [9, 13]. However, the criteria to decide when patients with TS should undergo thyroidectomy are lacking. There are few reported cases of TS treated by thyroidectomy alone; and in another instance, ECMO was used in a patient who responded to the medical treatment but not both together. We searched the literature for a clear definition of the refractory TS as an `indication for surgery', but we could not reach an explicit answer. In our cases, we could not determine the optimum timing to proceed with surgery. Whether early surgery can improve the outcomes in terms of hospital length of stay, numbers of failing organs, and ECMO-related costs and complications; this question needs thorough discussion and consensus.

Multiorgan failure, including hepatic dysfunction, may be one of the significant complications. It could be related to antithyroid medications, heart failure or the TS itself [6]. Hepatic dysfunction played the main role to proceed with surgery as it limited the antithyroid medications use. Therefore, for patients with TS complicated by organ failure refractory to intensive medical therapy, timely surgery needs to be considered. Surgery represents a valid intervention to control the source of the storm. It also sheds light on the role of ECMO as a bridge to a safe surgery.

## CONCLUSIONS

Precautions concerning the BB and antithyroid medications use in TS, especially in acute settings, should be considered. BB may precipitate cardiovascular collapse due to inhibition of thyroid-induced hyperadrenergic compensation, which maintains cardiac output in these patients. Upon its availability, the use of ECMO and early thyroidectomy is efficient in patients not responding to medical treatment.

## AUTHORS’ CONTRIBUTIONS

All authors have substantial contribution and have read and approved this manuscript and submission.

## Supplementary Material

CARE_Checklist_R_rjac131
