# Supplementary material for: Extracorporeal membrane oxygenation support and total thyroidectomy in patients with refractory thyroid storm: case series and literature review
Source: J Surg Case Rep. 2022 May 17;2022(5):rjac131. doi: 10.1093/jscr/rjac131 (PMC9113021; doi:10.1093/jscr/rjac131)
Supplement: CARE_Checklist_R_rjac131 [file care_checklist_r_rjac131.docx]

| **Topic** | **Item** | **Checklist item description** | **Reported on page** |
| --- | --- | --- | --- |
| **Title** | **1** | The words “case report” should be in the title along with what is of greatest interest in this case | 1 |
| **Key Words** | **2** | The key elements of this case in 2 to 5 key words | 2 |
| **Abstract** | **3a** | Introduction—What is unique about this case? What does it add to the medical literature? | 2 |
|  | **3b** | The main symptoms of the patient and the important clinical findings | 2 |
|  | **3c** | The main diagnoses, therapeutics interventions, and outcomes | 2 |
|  | **3d** | Conclusion—What are the main “take-away” lessons from this case? | 2 |
| **Introduction** | **4** | Brief background summary of this case referencing the relevant medical literature | 3 |
| **Patient Information** | **5a** | Demographic information (such as age, gender, ethnicity, occupation) | 3 |
|  | **5b** | Main symptoms of the patient (his or her chief complaints) | 3-4 |
|  | **5c** | Medical, family, and psychosocial history including co-morbidities, and relevant genetic information | 3-4 |
|  | **5d** | Relevant past interventions and their outcomes | n/a |
| **Clinical Findings** | **6** | Describe the relevant physical examination (PE) findings | 3-4 |
| **Timeline** | **7** | Depict important milestones related to your diagnoses and interventions (table or figure) | 3-4 and fig/tab |
| **Diagnostic Assessment** | **8a** | Diagnostic methods (such as PE, laboratory testing, imaging, questionnaires) | 3-4 and fig/tab |
|  | **8b** | Diagnostic challenges (such as financial, language, or cultural) | n/a |
|  | **8c** | Diagnostic reasoning including other diagnoses considered | 3-4 and fig/tab |
|  | **8d** | Prognostic characteristics (such as staging in oncology) where applicable | n/a |
| **Therapeutic Intervention** | **9a** | Types of intervention (such as pharmacologic, surgical, preventive, self-care) | 4-5 |
|  | **9b** | Administration of intervention (such as dosage, strength, duration) | 4-5 |
|  | **9c** | Changes in intervention (with rationale) | 4-5 |
| **Follow-up and Outcomes** | **10a** | Clinician-assessed outcomes and when appropriate patient-assessed outcomes | 4-5 |
|  | **10b** | Important follow-up test results | 5 |
|  | **10c** | Intervention adherence and tolerability (How was this assessed?) | Figures/tab |
|  | **10d** | Adverse and unanticipated events | 4-5 |
| **Discussion** | **11a** | Discussion of the strengths and limitations in the management of this case | 5-7 |
|  | **11b** | Discussion of the relevant medical literature | 5-7 |
|  | **11c** | The rationale for conclusions (including assessment of possible causes) | 5-7 |
|  | **11d** | The main “take-away” lessons of this case report | 7 |
| **Patient Perspective** | **12** | Did the patient share his or her perspective or experience? (Include when appropriate) | n/a |
| **Informed Consent** | **13** | Did the patient give informed consent? Please provide if requested:  Data and images (laboratory readings) are entirely unidentifiable and there are no details on individuals reported within the manuscript, consent for publication of images was not required and accordingly the medical research center approve this case report (IRB: MRC-04-21-907) | **Yes _ __ No _X__** |

**CARE Checklist (2013) of information to include when writing a case report**
